# Denoising Diffusion Probabilistic Models and Transfer Learning for citrus disease diagnosis

**DOI:** 10.3389/fpls.2023.1267810

**Published:** 2023-12-11

**Authors:** Yuchen Li, Jianwen Guo, Honghua Qiu, Fengyi Chen, Junqi Zhang

**Affiliations:** School of Mechanical Engineering, Dongguan University of Technology, Dongguan, Guangdong, China

**Keywords:** plant disease diagnosis, citrus, Denoising Diffusion Probabilistic Models (DDPM), Transfer Learning, Swin Transformer

## Abstract

**Problems:**

Plant Disease diagnosis based on deep learning mechanisms has been extensively studied and applied. However, the complex and dynamic agricultural growth environment results in significant variations in the distribution of state samples, and the lack of sufficient real disease databases weakens the information carried by the samples, posing challenges for accurately training models.

**Aim:**

This paper aims to test the feasibility and effectiveness of Denoising Diffusion Probabilistic Models (DDPM), Swin Transformer model, and Transfer Learning in diagnosing citrus diseases with a small sample.

**Methods:**

Two training methods are proposed: The Method 1 employs the DDPM to generate synthetic images for data augmentation. The Swin Transformer model is then used for pre-training on the synthetic dataset produced by DDPM, followed by fine-tuning on the original citrus leaf images for disease classification through transfer learning. The Method 2 utilizes the pre-trained Swin Transformer model on the ImageNet dataset and fine-tunes it on the augmented dataset composed of the original and DDPM synthetic images.

**Results and conclusion:**

The test results indicate that Method 1 achieved a validation accuracy of 96.3%, while Method 2 achieved a validation accuracy of 99.8%. Both methods effectively addressed the issue of model overfitting when dealing with a small dataset. Additionally, when compared with VGG16, EfficientNet, ShuffleNet, MobileNetV2, and DenseNet121 in citrus disease classification, the experimental results demonstrate the superiority of the proposed methods over existing approaches to a certain extent.

## 1 Introduction

Early detection of crop disease symptoms is a vital means of protecting crops and containing outbreaks (Thomas et al., 2018). Machine vision provides an intuitive and visual representation of crop growth, fruit quality, maturity, and can accurately identify healthy crops, diseased crops, and the types of pathogens (Sankaran et al., 2010; Jahanbakhshi et al., 2021; Momeny et al., 2022; Azadnia et al., 2023; Hadipour-Rokni et al., 2023). Throughout the various stages of crop cultivation, plant diseases often manifest in the leaves, making leaf disease identification critically important (Kailasam et al., 2022).

Research has been conducted on the automatic recognition of plant disease leaf images using machine learning techniques. Hossain et al. (2019) proposed a method for detecting and characterizing plant leaf diseases using KNN classifiers. Gupta et al. (2021) introduced a machine learning-based intelligent optimization algorithm to handle noise in dataset for plant leaf disease diagnosis. Zhu et al. (2017) proposed a hyperspectral imaging method for pre-detecting tobacco disease symptoms based on continuous projection algorithm and machine learning classifiers. Iniyan et al. (2020) utilized Support Vector Machines and Artificial Neural Networks for plant disease recognition and detection. Bhatia et al. (2020) investigated the application of Extreme Learning Machines in predicting plant diseases in highly imbalanced dataset. Arora et al (Arora and Agrawal, 2020). developed a deep forest method for classifying maize plant leaf diseases.

The aforementioned research were based on shallow machine learning models, and their identification performance heavily depended on expert experience, which limited their generalization ability (Sujatha et al., 2021). In contrast, deep learning models can effectively reduce the interference of expert experience while ensuring recognition accuracy (Lee et al., 2020). Currently, mainstream methods are shifting towards the application of deep learning (Lee et al., 2020; Sujatha et al., 2021). Intelligent diagnostic methods based on deep learning mechanisms can effectively address complex input and classification problems and have been applied to establish intelligent models for disease and pest diagnosis in crops such as maize, wheat, citrus, and potatoes (Lee et al., 2020; Sujatha et al., 2021). However, the complex and dynamic agricultural growth environment results in significant variations in the distribution of state samples, with existing research mostly relying on laboratory public dataset, such as Plantvillage (Hughe and Salathé, 2015). The scarcity of real disease databases weakens the information carried by the samples (Arnal, 2018), posing higher requirements for establishing deep learning intelligent diagnosis models.

In recent years, the combination of diffusion models and the Swin Transformer model has proven to be highly effective in small sample application environments, yielding satisfactory results. Inspired by non-equilibrium thermodynamics, the Denoising Diffusion Probabilistic Models (DDPM) (Ho et al., 2020) define a Markov diffusion step chain, where each diffusion step depends solely on the data distribution state of the previous step. Compared to Generative Adversarial Networks (GANs), DDPM offers more stable training and can generate more diverse samples (Croitoru et al., 2023). The Self-Attention Mechanism (SAM) (Yang, 2020; Pan et al., 2022) is widely used in various fields of artificial intelligence and has successfully boosted the performance of different models. Swin Transformer (Liu et al., 2021) introduces a hierarchical transformer structure, giving the transformer a layered structure similar to Convolutional Neural Networks (CNNs), with multi-scale features. Swin Transformer has achieved promising results in object recognition tasks on datasets such as CIFAR-10, CIFAR-100, SVHN, and ImageNet (Lee et al., 2021).

This paper establishes a practical citrus disease database and proposes two methods to test the effectiveness of diffusion models and the Swin Transformer model in diagnosing citrus diseases with small-sample. Furthermore, we compare the method 2 with various deep learning approaches, and the results indicate certain advantages of the proposed methods.

The subsequent organization of this paper includes: the second part, which presents related research; the third part, explaining the principles and the two proposed methods; the fourth part, which covers the experiments and discussions; and the final part, providing conclusions and future work.

## 2 Related research

Deep learning models can effectively reduce the reliance on expert experience while ensuring satisfactory recognition performance. In recent years, there has been much research in the field of intelligent diagnosis utilizing deep learning. Sujatha et al. (2021) compared various machine learning and deep learning methods for plant disease detection, such as Support Vector Machines (SVM), Random Forest (RF), and deep learning models like Inception-v3, VGG-16, and VGG-19. Their experimental results showed that deep learning outperformed machine learning methods in citrus plant disease detection accuracy. Zhang et al. (2019) proposed a cucumber leaf disease recognition method based on CNN. Geetharamani et al (Geetharamani and Pandian, 2019). employed a nine-layer deep CNN for plant leaf disease recognition. Tang et al. (2020) utilized CNN for grape disease image classification. Agarwa et al (Agarwal et al., 2020). developed an Efficient CNN model for tomato crop disease recognition. Sathiand et al (Dananjayan et al., 2022). studied advanced CNN detectors for citrus leaf disease detection and evaluated each model based on parameters such as accuracy and recall rate, finding that CenterNet2 and Res2Net-101-DCN-BiFPN achieved high-precision prediction of early citrus leaf diseases.

Few-shot learning and Transfer Learning were initially introduced within the context of applications with limited sample sizes. Argueso et al (Argüeso et al., 2020). studied Few-shot learning methods for plant disease classification using field-collected images. They employed Few-shot Learning algorithms to learn new plant leaf and disease types from extremely small dataset, achieving superior performance compared to classical learning methods while reducing training data by approximately 90%. Lee et al (Douarre et al., 2019). designed two new data generation methods, based on plant canopy simulation and GAN, to address the challenging segmentation task of apple scab disease in apple canopy images using CNN, obtaining satisfactory results on small dataset. Atila et al. (2021) proposed an efficient deep learning architecture for plant leaf disease classification, using Transfer Learning to train EfficientNet and other deep learning models. Jiang et al. (2021) improved the VGG16 model based on multi-task learning for the identification of three types of rice leaf diseases and two types of wheat leaf diseases, using pre-trained models from ImageNet for transfer learning, resulting in simultaneous recognition of rice and wheat leaf diseases and providing a reliable method for identifying multiple plant leaf diseases. Chen et al. (2020) studied Transfer Learning with deep CNNs for identifying plant leaf diseases, considering using pre-trained models learned from massive dataset and then transferring them to specific tasks. Compared to other methods, their validation accuracy on public dataset was not lower than 91.83%. Even under complex background conditions, their method achieved an average classification prediction accuracy of 92.00% on rice plant images.

While deep learning and its related techniques have demonstrated impressive results in the diagnosis of plant diseases, obtaining a sufficient number of disease samples continues to be a challenge. This difficulty hampers the development of robust deep diagnostic models. Moreover, employing models optimized in a controlled laboratory setting proves ineffective in real-world scenarios due to the challenge of meeting the independent and identically distributed condition between experimental data and practical application data. Addressing these challenges and harnessing the full potential of deep learning mechanisms to create intelligent diagnostic models tailored for agricultural applications represents a crucial problem that needs to be addressed.

## 3 Principles and methods

### 3.1 DDPM

**Figure 1:**
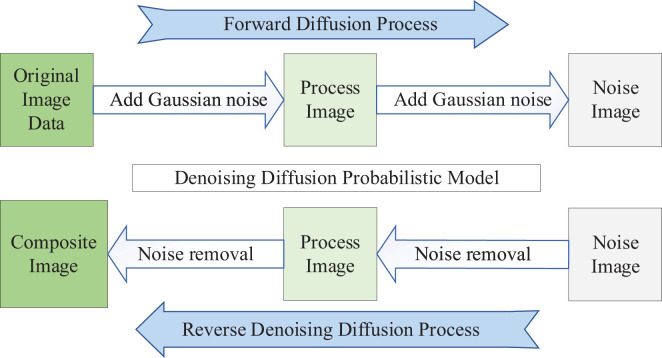
DDPM.

The DDPM (Sohl-Dickstein et al., 2015) can be used as a data augmentation technique to increase the size of the dataset and prevent overfitting of the network. DDPM consists of two processes: the Forward Diffusion Process and the Reverse Denoising Diffusion Process. Both processes are parameterized Markov Chains. The essence of the DDPM diffusion model is learning a “denoising” process, as shown in **Figure 1**. From an individual image’s perspective, the Forward Diffusion Process gradually adds Gaussian Random Noise to the image until it becomes a pure noise image. On the other hand, the Reverse Denoising Diffusion Process generates an image from a pure noise image. By training the DDPM diffusion model to learn the diffusion process of the image data, when properly trained, random noise images are input into the DDPM. The Reverse Denoising Diffusion Process is executed, gradually “denoising” the pure noise image, resulting in a synthesized image similar to the real image.

### 3.2 Transfer Learning

Transfer Learning (Zhuang et al., 2020) is a training method that involves transferring the network architecture and weights originally used for solving task A to task B, and achieving good results in task B as well. In scenarios with a small sample size, Transfer Learning can be employed to transfer the learned generic features from other pre-trained networks, saving training time and obtaining better recognition results. Fine-Tuning (Too et al., 2019) is a commonly used implementation approach within transfer learning. Fine-Tuning preserves the existing network architecture and pre-trained model parameters while retraining, making minor adjustments to the model parameters. It does not involve pruning and reconstructing network parameters, making it a holistic, global, and subtle improvement.

In this study, we used Transfer Learning with Fine-Tuning, implemented as follows: we imported pre-trained weights into the classification model, removed the weights related to the fully connected layer for classification, retained the other weights in the model, and did not freeze the weight parameters. We modified the model’s classification output categories (from 1000 to 3), and trained the modified classification model with the training data in batches. This process resulted in retraining the weights of the fully connected layer and fine-tuning the other pre-trained weight parameters in the model.

### 3.3 Self-attention mechanism

The SAM (Vaswani et al., 2017) is a neural network architecture that allows the computer to automatically learn and focus on the most important information when processing input data, thus improving its processing efficiency and reducing the time spent in noise. The implementation of SAM can be divided into three steps: (1) Building the attention layer, which uses learnable parameters to measure the importance of input information; (2) Mapping the input data and the attention layer’s parameters to the output information; (3) Calculating the loss function and updating the parameters of the attention layer to enable better focus on the most important information.

**Figure 2:**
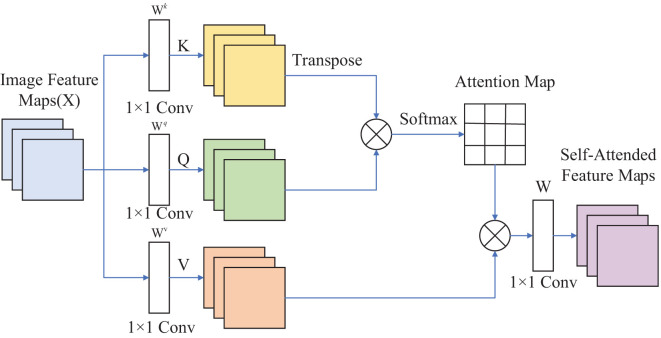
Self-attention mechanism.


**Figure 2** illustrates the application of SAM to an image. The self-attention computation for input feature maps, denoted as Image Feature Map X, can be expressed as shown in Equation 1.

(1) $$\text{A}ttention(Q,K,V)=SoftMax(\frac{QK^{T}}{\sqrt{\text{d}}})V$$

Where *Q*, *K*, and *V* represent *Query*, *Key*, and *Value*, respectively. *Query* can be considered as the Question, *Key* as the Index, and *Value* as the Answer. $\text{Q}=W^{q}•I$ , $\text{K}=W^{k}•I$ , and $V=W^{v}•I$ is the vector corresponding to the position on the input matrix *X*. Reshape *X* into three matrices: *K*, *Q*, and *V*. Compute the product of the Transpose of *K* and *Q*, then divide the result by $\sqrt{d}$ . Apply the Softmax Function to normalize the values and obtain the Attention Map. Finally, multiply the Attention Map by *V* and reshape it using *W* to obtain the output feature maps, known as *Self-Attended Feature Maps*.

### 3.4 Swin Transformer

**Figure 3:**
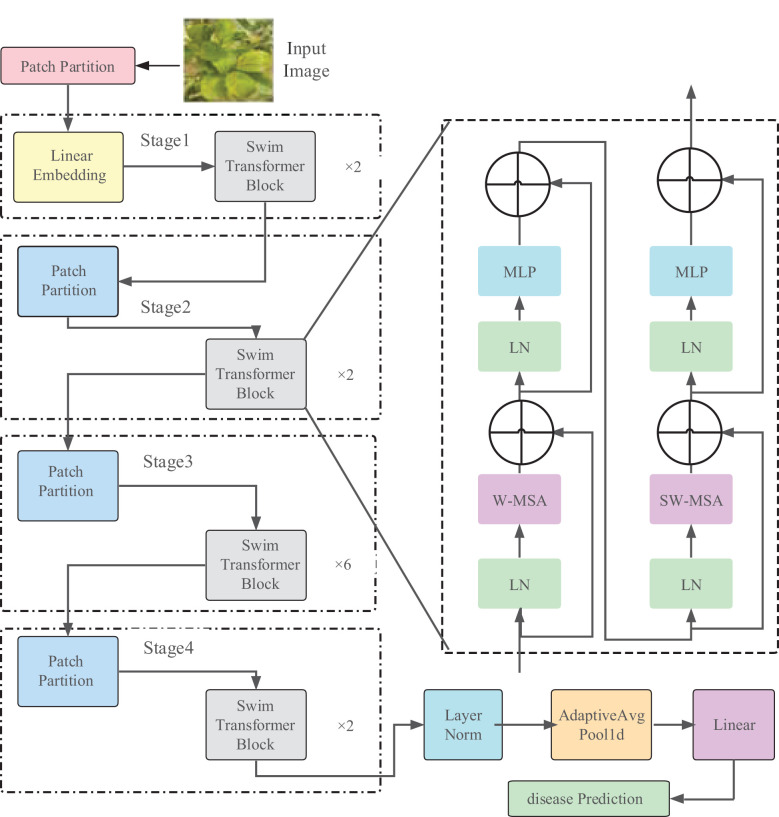
**D**iagram of Swin Transformer (tiny) network architecture.

The Swin Transformer model is illustrated in **Figure 3**, and the model parameters are shown in **Table 1**. As an instance of the ‘encoder-decoder’ architecture of the Transformer, its encoder and decoder consist of stacked modules based on self-attention. The embeddings of the source (input) sequence and the target (output) sequence are augmented with positional encoding and then separately fed into the encoder and decoder. The Swin Transformer introduces a hierarchical structure, which differs from the standard Transformer architecture, as it computes non-overlapping windows for self-attention. This endows the Transformer with a hierarchical structure similar to CNN, providing multi-scale features and better applicability in downstream tasks.

**Table 1:** Parameters of the Swin-T (tiny) network architecture.

Stage	Dowmsp.rate(output size)	Swim-T(tiny)
Stage1	4×(56×56)	Concat4×4,96−d,LN
		$\left[\begin{array}{cc} \text{win}.sz. & 7\times 7\\ \dim96 & \text{head }3 \end{array}\right]\times 2$
Stage2	8×(28×28)	Concat2×2,192−d,LN
		$\left[\begin{array}{cc} \text{win}.sz. & 7\times 7\\ \dim192 & \text{head }6 \end{array}\right]\times 2$
Stage3	16×(14×14)	Concat2×2,384−d,LN
		$\left[\begin{array}{cc} \text{win}.sz. & 7\times 7\\ \dim384 & \text{head }12 \end{array}\right]\times 6$
Stage4	32×(7×7)	Concat2×2,768−d,LN
		$\left[\begin{array}{cc} \text{win}.sz. & 7\times 7\\ \dim768 & \text{head }24 \end{array}\right]\times 2$

**Figure 4:**
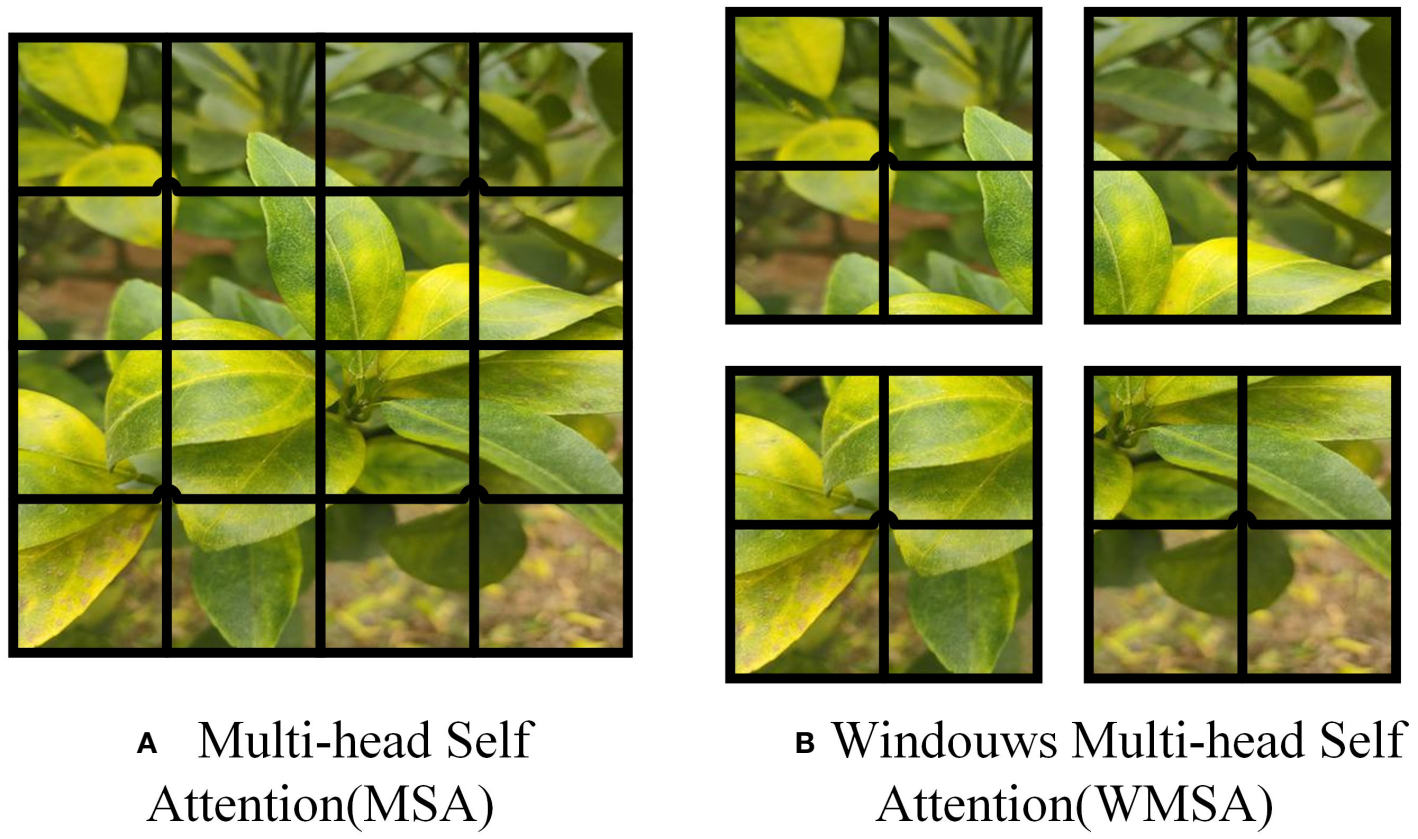
W-MSA example.

In the Swin Transformer network architecture, the Swin Transformer Block employs Windows Multi-Head Self-Attention (W-MSA) (Li et al., 2021) and Shifted Window Multi-Head Self-Attention (SW-MSA) (Han et al., 2023). The purpose of W-MSA is to reduce computational complexity. As illustrated in **Figure 4**, **Figure 4A** depicts a standard Multi-Head Self-Attention (MSA) (Rao et al., 2021), where each pixel (or token, patch) in the Feature Map needs to compute attention with all other pixels during the Self-Attention process. In **Figure 4B**, when utilizing W-MSA, the Feature Map is initially divided into separate windows of size M*M (where M=2 in the example), and then Self-Attention is independently calculated within each window.

**Figure 5:**
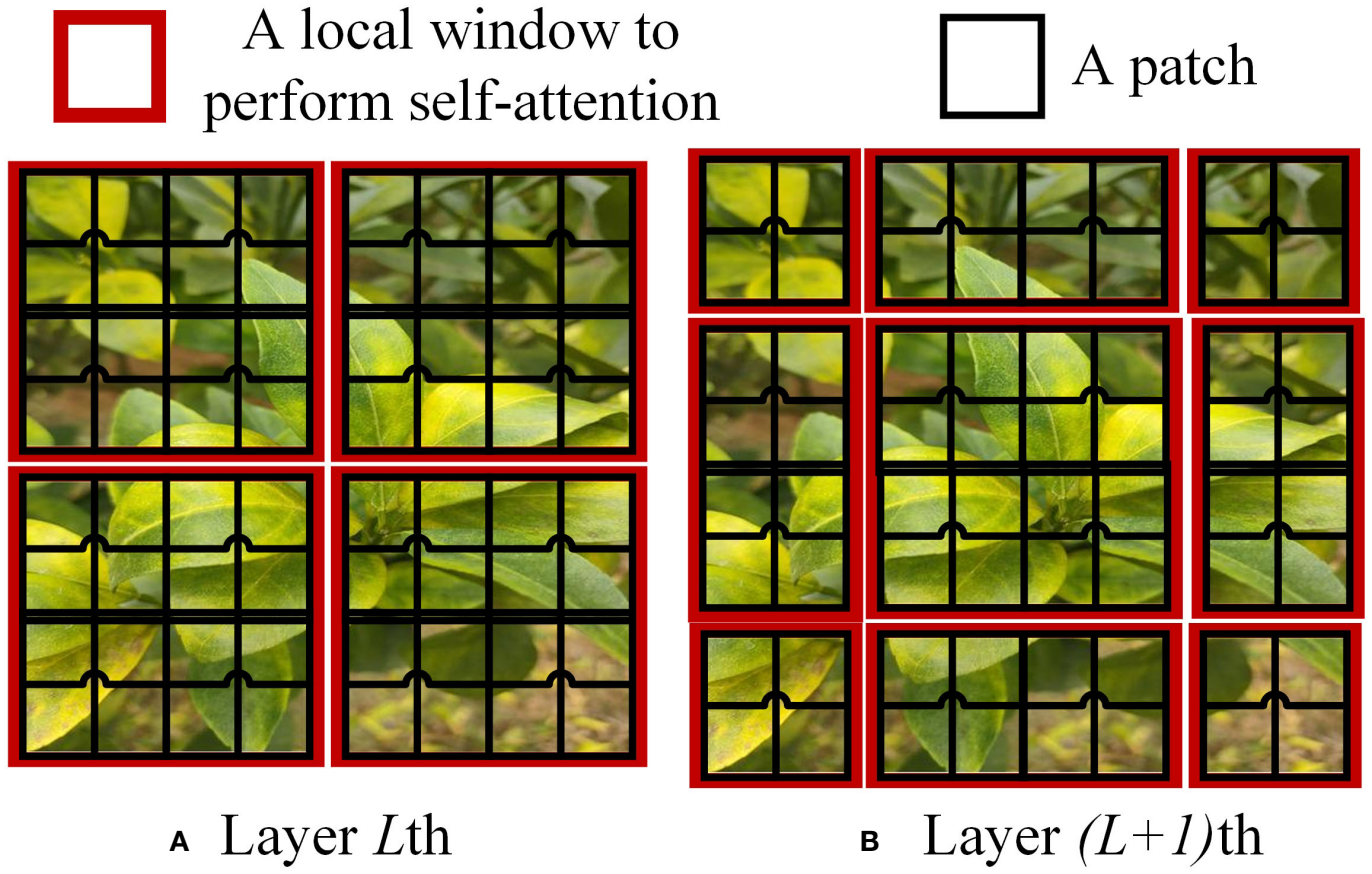
SW-MSA example.

When using the W-MSA module, Self-Attention calculations are performed only within each window, and there is no information exchange between different windows. To address this issue, Swin Transformer introduces SW-MSA. As shown in **Figure 5A**, W-MSA and SW-MSA are used in pairs. W-MSA is used in the *L*th layer, and since W-MSA and SW-MSA are used in pairs, the *(L+1)*th layer uses SW-MSA, as shown in **Figure 5B**. In **Figure 5A**, windows have been shifted, and by comparing the left and right diagrams, it can be observed that the windows have shifted. For example, the 2x4 window in the first row and second column can facilitate information exchange between the two windows in the *L*th layer; similarly, the 4x4 window in the second row and second column can facilitate information exchange between the four windows in the *L*th layer, and so on for others. This effectively solves the problem of no information exchange between different windows.

### 3.5 Method

In this study, we established our own practical on-site citrus disease database to test the feasibility and effectiveness of diagnosing citrus diseases with small samples. Two testing methods were employed.

#### 3.5.1 The Method 1

**Figure 6:**
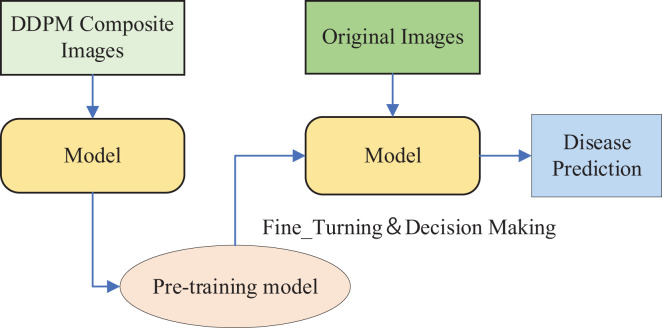
The Method 1.

The DDPM model was used to generate synthetic images for data augmentation, and the Swin Transformer model was pre-trained on the synthetic dataset generated by DDPM. Subsequently, Fine-Tuning was performed on the original citrus leaf images for disease classification using the pre-trained Swin Transformer model, as illustrated in **Figure 6**.

#### 3.5.2 The Method 2

**Figure 7:**
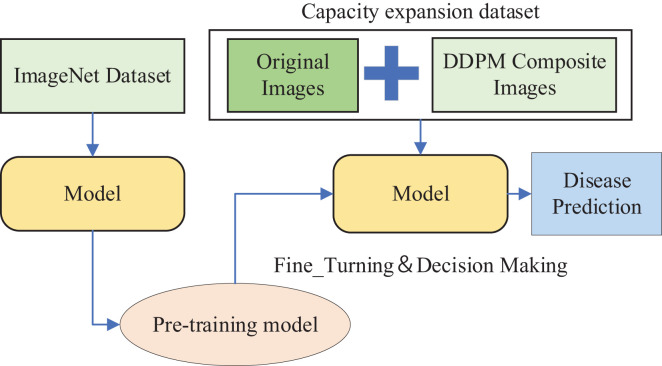
The Method 2.

The DDPM model was used to generate synthetic images for data augmentation. We utilized the pre-trained Swin Transformer model on the ImageNet dataset (Deng et al., 2009) and performed Transfer Learning by Fine-Tuning it on an expanded dataset composed of the original dataset and the synthetic images generated by DDPM, as depicted in **Figure 7**.

## 4 Experiment and discussion

### 4.1 Dataset preparation

**Figure 8:**
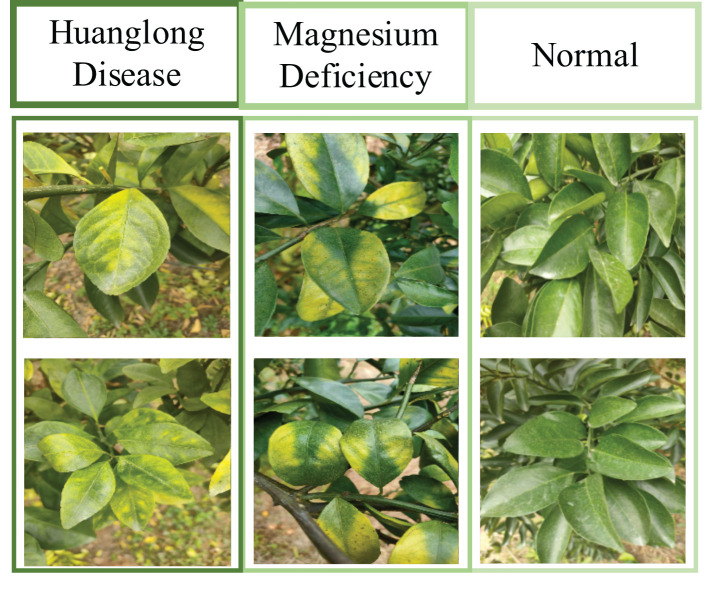
Example of citrus dataset images.

The original dataset used in this project was a citrus image dataset established by the project team. **Figure 8** is an example of Citrus Dataset Images. The dataset has 2,648 images and consists of three categories: Huanglongbing-infected leaves (758 images), Magnesium-deficient leaves (739 images), and Healthy leaves (1,151 images). The images are in the format of 4000 * 3000 * 3. The citrus image dataset was collected in the field under adaptive photography mode, making it more suitable for practical application environments.

### 4.2 Algorithm performance metrics

The performance of the proposed methods was evaluated using nine performance metrics: Accuracy, Precision, Recall, F1 score, F2 Score, Specificity, Matthews correlation coefficient (MCC), True Positive Rate (TPR), and False Positive Rate (FPR). The descriptions of these performance metrics are shown in **Table 2**.

**Table 2:** Performance metrics.

Performance Index	Formula
Accuracy	$\frac{TP+TN}{TP+TN+FP+FN}$
Precision	$\frac{TP}{TP+FP}$
Recall	$\frac{TP}{TP+FN}$
F1 Score	$\frac{2TP}{2TP+FN+FP}$
F2 Score	$\frac{5\times \text{Accuracy}\times \text{Recall}}{4\times \text{Accuracy}+\text{Recall}}$
Specificity	$\frac{TN}{TN+FP}$
MCC	$\frac{TP\times TN-FP\times FN}{\sqrt{\left(TP+FP\right)\left(TP+FN\right)\left(TN+FP\right)(TN+FN)}}$
TPR	$\frac{TP}{TP+FN}$
FPR	$\frac{FP}{FP+TN}$

TP (True Positive) refers to the cases where the true class is positive, and the predicted class is also positive; FP (False Positive) refers to the cases where the true class is negative, but the predicted class is positive; FN (False Negative) refers to the cases where the true class is positive, but the predicted class is negative; TN (True Negative) refers to the cases where the true class is negative, and the predicted class is also negative.

**Figure 9:**
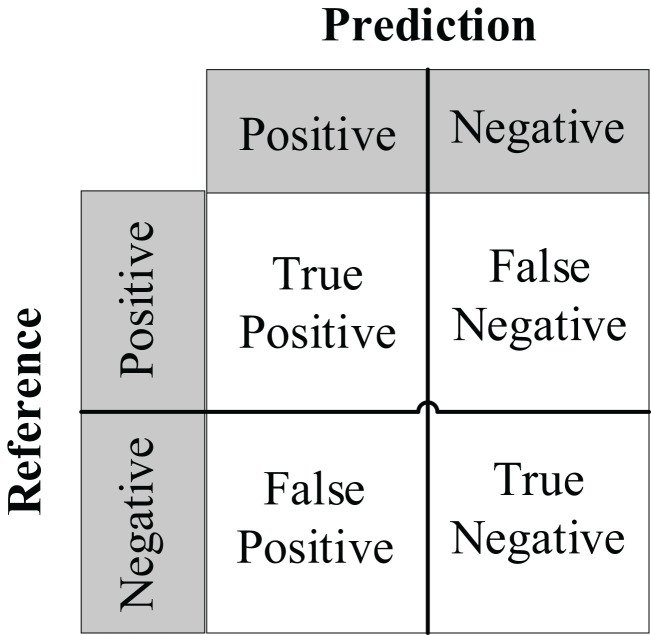
Confusion matrix.

We use Confusion Matrix (Görtler et al., 2022) as a visualization tool to compare the classification results with the actual values. As shown in **Figure 9**, each column of the Confusion Matrix represents the predicted class, and the total count in each column indicates the number of data instances predicted as that class. Each row represents the true class of the data, and the total count in each row indicates the number of data instances belonging to that class. The Receiver Operating Characteristic Curve (ROC Curve) is a graphical analysis tool used to determine the optimal threshold within the same classifier model. The ROC Curve is a plot with the False Positive Rate (FPR) on the x-axis and the True Positive Rate (TPR) on the y-axis, allowing the classifier to be mapped to a point on the ROC plane (FPR, TPR). By adjusting the threshold used for classification in this classifier, a curve passing through points (0, 0) and (1, 1) can be obtained, which is the ROC Curve for that classifier. The Area Under the Curve (AUC) is defined as the area under the ROC Curve, and a higher AUC value, closer to 1.0, indicates greater classifier accuracy.

### 4.3 Experimental configuration

#### 4.3.1 Experimental environment

The experiments were conducted on a Lenovo R7000P2020 edition device (running Windows 11 with an AMD Ryzen 7 4800H processor and an RTX 2060 6GB graphics card). The Python environment used was Anaconda3 (Python 3.7), with Torch 1.9.0, Torchvision 0.10.0, and OpenCV 4.5.1 installed. The training process was accelerated using the GPU.

#### 4.3.2 Data classification

The experiment utilized a citrus leaf dataset for training. The dataset was split into training (train), validation (val), and testing (test) sets in an 8:1:1 ratio. The input images were normalized to 224 x 224 x 3 and fed into the neural network for training and evaluation. During the training process, the training and validation sets were used, while the testing set was utilized for subsequent performance testing.

#### 4.3.3 Parameter settings

The training process employed the SGDM optimizer with L2 regularization. The momentum was set to 0.9, and the weight decay was set to 5E-5. The batch_size was 16, and num workers was set to 0. The Initial Learning Rate was set to 0.001, and the Cosine Annealing Learning Rate adjustment strategy was used. The Minimum Learning Rate was set to 0, and the learning rate was reduced in a cosine manner over 100 epochs.

### 4.4 Algorithm performance experiments

The Algorithm Performance Experiments aimed to test and evaluate the performance of the proposed methods, and were divided into four experiments:

Experiment 1: DDPM Synthetic Citrus Leaf Dataset Generation. The DDPM model was trained on the original citrus leaf dataset, and the model’s fitting effect was evaluated and the weights were updated after each training epoch to produce synthetic images that closely resembled real images. After the network training was completed, the DDPM model was used to generate synthetic citrus leaf images for each of the three categories, resulting in a total of 1000 synthetic images for each citrus leaf category to form the synthetic citrus leaf dataset.
Experiment 2: Swin-T (Orgin). The Swin Transformer model was trained on the original citrus dataset for 100 epochs without utilizing any pre-trained weight models, serving as the control group.
Experiment 3: Swin-T (DDPM data Pre-train model + Orgin data). The Swin Transformer model was initially pre-trained using the DDPM synthetic citrus leaf dataset and subsequently fine-tuned on the original citrus leaf dataset. The pre-training on the DDPM synthetic dataset was conducted for 100 epochs, followed by fine-tuning on the original dataset for an additional 100 epochs. This experiment employed the method proposed in this study.
Experiment 4: Swin-T (ImageNet data Pre-train model + Expanded data). The Swin Transformer model, pre-trained on the ImageNet dataset, was fine-tuned on the extended dataset comprising the original citrus leaf dataset and the DDPM synthetic dataset. The pre-trained model from the ImageNet dataset was transferred to the extended dataset for fine-tuning, which was conducted for 100 epochs. This experiment also employed the method proposed in this study.

The comparison of accuracy between the Experiment 2 and the Experiment 3 aimed to verify the superiority of the proposed Method 1 in the training process. Similarly, the comparison of accuracy between the Experiment 2 and the Experiment 4 aimed to verify the superiority of the proposed Method 2.

**Figure 10:**
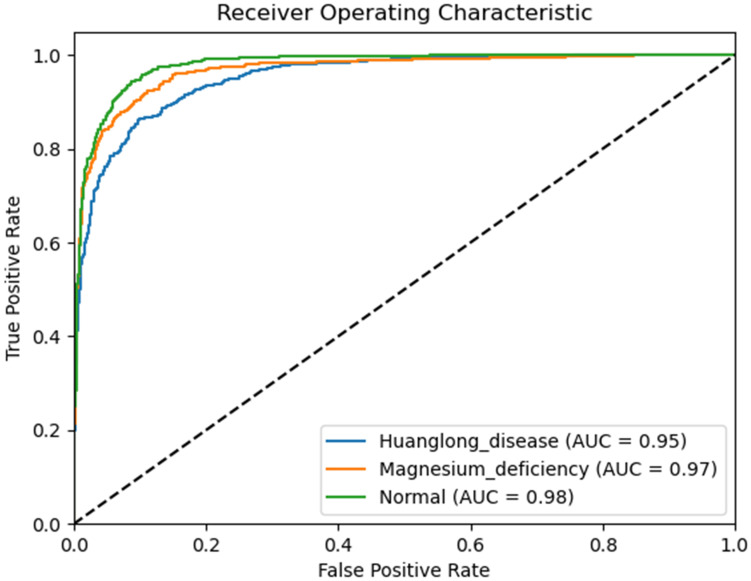
ROC Curve of the Experiment 2.

**Figure 11:**
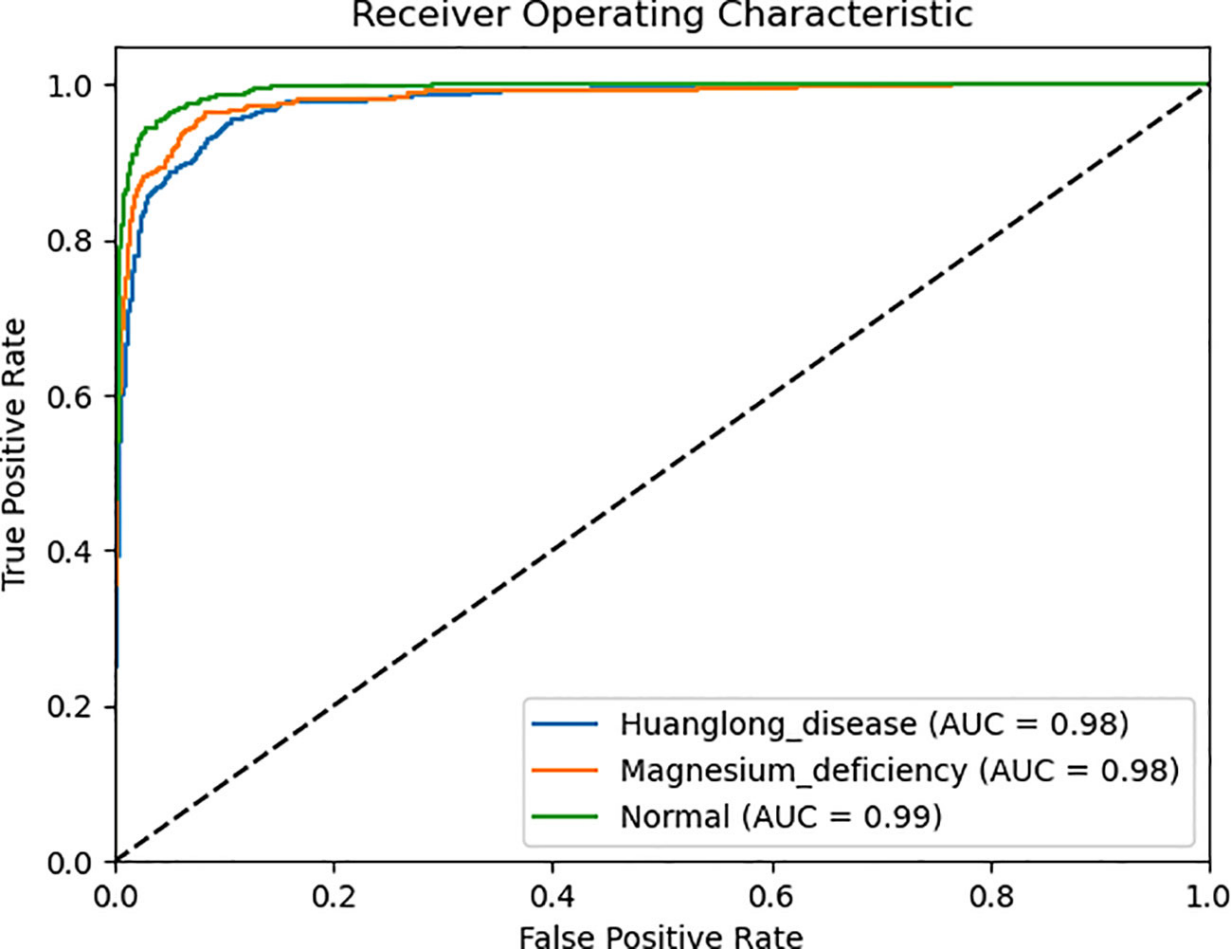
ROC Curve of the Experiment 3.

**Figure 12:**
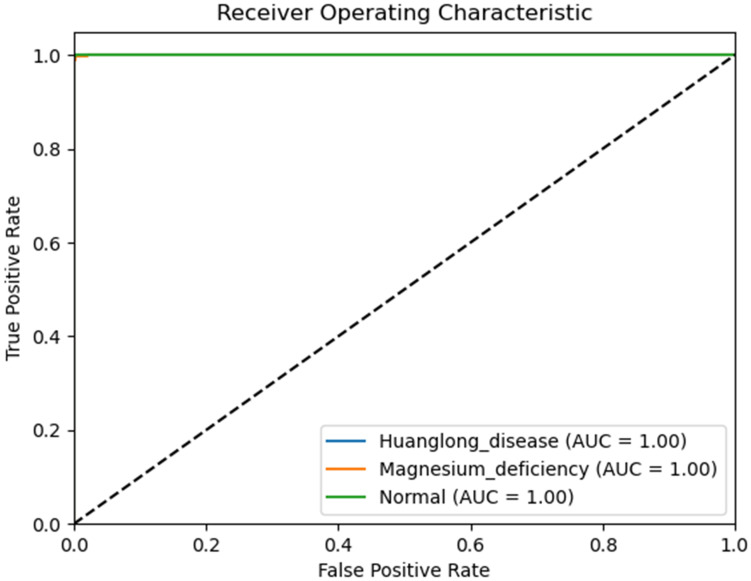
ROC curve of the Experiment 4.

**Figure 13:**
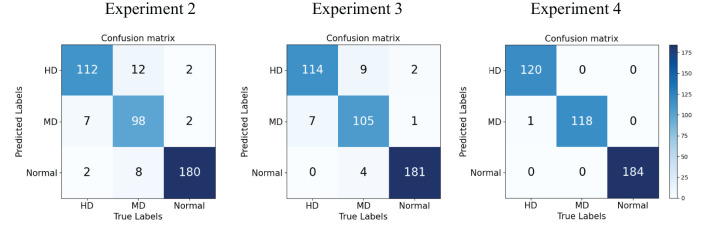
Confusion matrix of the Experiment 2-4.

**Figure 14:**
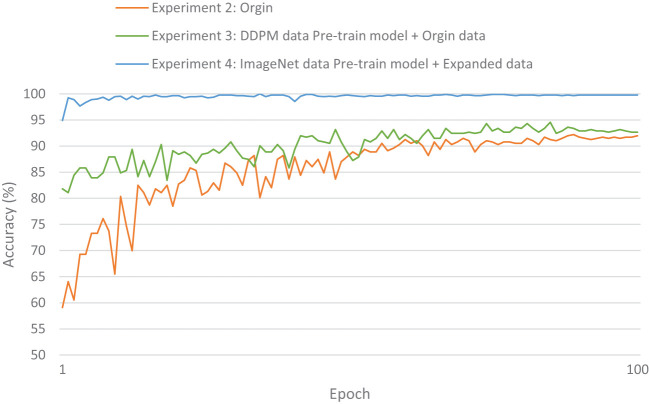
Validation accuracy of the Experiment 2-4.

**Figure 15:**
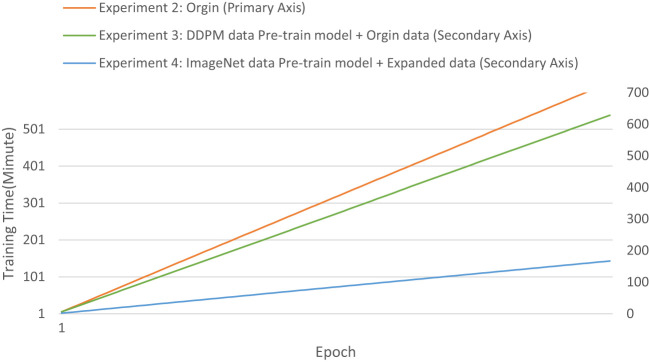
The cumulative training time for each epoch of the Experiment 2-4.

The evaluation metrics for different disease categories in the Experiments 2, 3, and 4 are shown in **Tables 3**–**5**, respectively. In the three different training approaches, Swin-T(Original), Swin-T (DDPM data Pre-train model + Original data), and Swin-T(ImageNet data Pre-train model + Expanded data), the ROC Curves are shown in **Figures 10**–**12**, respectively. The Confusion Matrixs are shown in **Figure 13**. The training validation accuracy data curve is depicted in **Figure 14**, while the cumulative training time for each epoch is shown in **Figure 15**. We evaluated the performance of the proposed method for the citrus disease leaf classification task. **Table 6** represents the classification performance of the proposed method on the original citrus leaf dataset. The proposed method achieved the highest validation accuracy of 99.8% for citrus disease leaf classification. From **Table 6**, it can be observed that compared to the original dataset, the Swin Transformer model showed improvements in accuracy, precision, recall, F1 score, and specificity for all classes after using the expanded dataset composed of original citrus data and DDPM-generated synthetic data, along with transfer learning. This improvement in classification performance clearly indicates that data augmentation with the DDPM model and the Transfer Learning method effectively prevent overfitting of the network and enhance its generalization ability.

**Table 3:** Experiment 2: Results of Swin-T(orgin).

Data Type	Huanglong disease	Magnesium deficiency	Normal
Accuracy	0.946	0.931	0.967
Precision	0.889	0.916	0.947
Recall	0.926	0.831	0.978
F1 Score	0.936	0.878	0.972
F2 Score	0.930	0.849	0.976
Specificity	0.954	0.970	0.958
MCC	0.869	0.826	0.933

**Table 4:** Experiment 3: Results of Swin-T(DDPM data Pre-train model + Orgin data).

Data Type	Huanglong disease	Magnesium deficiency	Normal
Accuracy	0.957	0.950	0.983
Precision	0.912	0.929	0.978
Recall	0.942	0.890	0.984
F1 Score	0.949	0.919	0.983
F2 Score	0.945	0.901	0.984
Specificity	0.964	0.974	0.983
MCC	0.897	0.875	0.966

**Table 5:** Experiment 4: Results of Swin-T(ImageNet data Pre-train model + Expanded data).

Data Type	Huanglong disease	Magnesium deficiency	Normal
Accuracy	0.998	0.998	0.999
Precision	0.999	0.992	0.999
Recall	0.992	0.999	0.999
F1 Score	0.995	0.999	0.999
F2 Score	0.993	0.999	0.999
Specificity	0.996	0.996	0.999
MCC	0.994	0.994	0.999

**Table 6:** Evaluation results of the three different training methods.

Model	Swin-T(orgin)	Swin-T (DDPM data Pre-train model + Orgin data)	Swin-T (ImageNet data Pre-train model + Expanded data)
Accuracy	0.948	0.963	0.998
Precision	0.917	0.940	0.997
Recall	0.912	0.939	0.997
F1 Score	0.928	0.950	0.998
F2 Score	0.918	0.943	0.997
Specificity	0.961	0.974	0.997
MCC	0.876	0.912	0.995

We evaluated the training speed of the proposed methods for citrus disease leaf classification tasks, as shown in **Table 7**, **Figure 14**, and **Figure 15**. In the Experiment 2, employing the Orgin training approach, the average time per epoch was 6.337 minutes. The initial validation accuracy (epoch 1) was 0.591, and the best validation accuracy (epoch 89) reached 0.948, with a total training duration of 559.162 minutes. In the Experiment 3, employing the Swin-T (DDPM data Pre-train model + Orgin data) training approach, the average time per epoch was 6.286 minutes. The initial validation accuracy (epoch 1) was 0.817, and the best validation accuracy (epoch 85) achieved 0.963, with a total training duration of 534.425 minutes. In the Experiment 4, employing the Swin-T (DDPM data Pre-train model + Orgin data) training approach, the average time per epoch was 1.671 minutes. The initial validation accuracy (epoch 1) was 0.949, and the best validation accuracy (epoch 35) reached 0.998, with a total training duration of 58.901 minutes. Comparatively, it was shown that the training time for the Swin-T (DDPM data Pre-train model + Orgin data) group is slightly shorter than that of the Orgin group, and significantly shorter than both the Swin-T (DDPM data Pre-train model + Orgin data) and Orgin groups. The experimental data clearly demonstrates that, on the Swin-T model, the first proposed method exhibits slightly faster training speed than the original training method, while the second proposed method exhibits significantly faster training speed.

**Table 7:** Time parameters of the Swin-T model under the three training methods.

Mode	Swin-T (orgin)	Swin-T (DDPM data Pre-train model + Orgin data)	Swin-T (ImageNet data Pre-train model + Expanded data)
Initial verification Accuracy	0.591	0.817	0.949
Best validation Accuracy	0.948	0.963	0.998
Average training time per epoch(minute)	6.337	6.286	1.671
Total time spent from initial to Best accuracy(minute)	559.162	534.425	58.901
Total epochs spent from initial to Best accuracy(Up to 100 epochs)	89	85	35

We evaluated the performance of the proposed methods for citrus disease leaf classification tasks. As depicted in **Table 8**, when compared to the original dataset, the Swin Transformer model demonstrated significant enhancements in various critical performance metrics, including accuracy, precision, recall, F1 score, F2 score, specificity, and MCC across all categories. These improvements were observed when the model was trained on an augmented dataset, which combined the original citrus dataset with a synthetic dataset generated by DDPM, followed by the application of transfer learning techniques. This notable enhancement in classification performance unequivocally signifies the effectiveness of data augmentation using the DDPM model and the transfer learning. These strategies not only mitigated overfitting but also bolstered the network’s capacity for generalization, underlining their crucial role in our approach.

**Table 8:** The performance of the three training methods.

Model	Swin-T(Orgin)	Swin-T (DDPM data Pre-train model + Orgin data)	Swin-T (ImageNet data Pre-train model + Expanded data)
Accuracy	0.948	0.963	0.998
Precision	0.917	0.940	0.997
Recall	0.912	0.939	0.997
F1 Score	0.928	0.950	0.998
F2 Score	0.918	0.943	0.997
Specificity	0.961	0.974	0.997
MCC	0.876	0.912	0.995

### 4.5 Algorithm performance comparison experiments

The algorithm performance comparison experiment involves applying the Swin Transformer, VGG16 (Xuemei et al., 2017), EfficientNet (Heidary-Sharifabad et al., 2021), ShuffleNet (Pani et al., 2019), MobileNetV2 (Dong et al., 2020), and DenseNet121 (Nandhini and Ashokkumar, 2022) models to the task of citrus disease classification. All six algorithm models undergo three sets of experiments.

Experiment 5 (Orgin): The six algorithm models are trained on the original dataset without using pre-trained weights, serving as the control group.

Experiment 6 (DDPM data Pre-train model + Orgin data): The six algorithm models are first pre-trained on the synthetic dataset generated by the DDPM model and then fine-tuned on the original citrus leaf dataset.

Experiment 7 (ImageNet data Pre-train model + Expanded data): The six algorithm models use their respective pre-trained models on the ImageNet dataset and then fine-tune on the expanded dataset consisting of the original citrus dataset and the DDPM synthetic dataset.

The experimental results are shown in **Table 9**. The Swin Transformer model achieves a validation accuracy of 94.8% in Experiment 5 (Orgin), ranking in the middle among the six trained models. In Experiment 6 (DDPM data Pre-train model + Orgin data), the Swin Transformer model achieves a validation accuracy of 96.3%, ranking the lowest among the six trained models. However, in Experiment 7 (ImageNet data Pre-train model + Expanded data), the Swin Transformer model achieves a validation accuracy of 99.8%, ranking first among all six training models, and achieving the highest rank in all experiments.

**Table 9:** Validation accuracy of different models under three training methods.

Methods	Orgin	DDPM data Pre-train model + Orgin data	ImageNet data Pre-train model + Expanded data
Swin-T(tiny)	0.948	0.963	0.998
Vgg16	0.957	0.992	0.990
EfficientNet_b0	0.969	0.985	0.995
ShuffleNet_v2_x0_5	0.931	0.964	0.988
MobileNetv2	0.990	0.985	0.995
Densenet121	0.988	0.997	0.990

### 4.6 Abalation experiments

Neural networks are also a black box system, and conducting ablation experiments can verify the connection between the proposed method and the component as a whole. Five Abalation experiments were conducted for each of the six different models, namely Swin-T, Vgg16, EfficientNet, ShuffleNet, MobileNetv2, and Densenet121, as outlined in **Table 10**. These experiments correspond to five training methods: Base, Base+A, Base+B, Base+C, and Base+B+C. Comparing the results between Base and Base+A reveals the effectiveness of Component A in improving overall accuracy. Likewise, the comparison between Base and Base+B demonstrates the impact of Component B on enhancing overall accuracy, and the comparison between Base and Base+C illustrates the contribution of Component C to overall accuracy improvement. Furthermore, by comparing the results of Base+A, Base+B, and Base+C, we can evaluate the performance differences among the three components in terms of their effect on overall accuracy improvement.

**Table 10:** Abalation experiments.

Methods	Dataset	Training method
Base	Orgin Dateset	Training from scratch
Base+A	Orgin Dateset	Fine-Tuning with DDPM pre-trained models
Base+B	Augmented dataset,	Training from scratch
Base+C	Orgin Dateset	Fine-Tuning with ImageNet pre-trained models
Base+B+C	Augmented dataset,	Fine-Tuning with ImageNet pre-trained models

A: Fine-Tuning with DDPM pre-trained models, B: using augmented dataset, C: Fine-Tuning with ImageNet pre-trained models.

As shown in **Table 11**, the comparisons of accuracy between Base and Base+A, Base+B, and Base+C across different models confirm that components A, B, and C individually contribute to improving model accuracy. For the Swin-T, EfficientNet_b0, ShuffleNet_v2_x0_5, and MobileNetv2 models, Component C has the most significant impact on improving accuracy. However, for the Vgg16 and Densenet121 models, Component A has the greatest influence on accuracy improvement. Notably, for the Vgg16 and Densenet121 models, the first proposed method (Base+A) achieves the highest model accuracy among the five groups. On the other hand, for the Swin-T, EfficientNet_b0, ShuffleNet_v2_x0_5, and MobileNetv2 models, the second proposed method (Base+B+C) attains the highest model accuracy among the five groups. It’s worth mentioning that in all five sets of experiments for each model, the Swin-T model in the Base+B+C group, using the Method 2 presented in this paper, achieves the best performance, reaching an impressive accuracy of 99.8%.

**Table 11:** Verifying accuracy of ablation experiments.

Methods	Base	Base+A	Base+B	Base+C	Base+B+C
Swim-T(tiny)	0.948	0.963	0.957	0.992	0.998
Vgg16	0.957	0.992	0.981	0.992	0.990
EfficientNet_b0	0.969	0.985	0.985	0.988	0.995
ShuffleNet_v2_x0_5	0.931	0.964	0.955	0.981	0.988
MobileNetv2	0.990	0.985	0.986	0.989	0.995
Densenet121	0.988	0.997	0.995	0.990	0.990

### 4.7 Discussion

In general, the CNN architecture has proven to be highly successful in visual tasks. It efficiently learns from samples by performing hard feature induction. The hierarchical structure of CNN is achieved through convolution and pooling operations, which capture local features in images and gradually abstract higher-level features. CNN models capture local context relationships through local receptive fields and parameter sharing. Compared to Transformers, CNNs generally have lower computational complexity and a stronger hierarchical structure.

The strong inductive bias of CNNs enables them to achieve high performance even with minimal data (high lower bounds). However, this same inductive bias may limit these models when abundant data is available (low upper bounds). As shown in the **Table 9**, CNN architectures such as VGG16, EfficientNet, ShuffleNet, MobileNetV2, and DenseNet121 generally outperform the Swin Transformer model in Experiment 5 (Orgin) and Experiment 6 (DDPM data Pre-train model + Orgin data) in terms of accuracy.

Visual models based on self-attention mechanisms do not perform well with small-scale data (low lower bounds) but have the potential to surpass CNN performance on large-scale datasets (high upper bounds). Unlike CNNs, self-attention-based visual models can capture global relationships between image elements and have stronger representational capabilities. However, Transformer architecture models need to learn this type of information from a large amount of data.

Since this study uses a small sample dataset, Swin Transformer’s performance on Experiment 5 and Experiment 6 with this dataset does not significantly outshine CNN models. However, when a pre-trained model from the large-scale ImageNet dataset is used and fine-tuning is performed with the original citrus dataset and DDPM synthetic dataset, the Swin Transformer achieves the best performance in Experiment 7 among all models. Therefore, when balancing factors such as dataset size, training approach, and the choice between CNN and Transformer models, decisions should be made based on the specific experimental conditions.

Both the Method 1 and the Method 2 proposed in this paper can be applied to both CNN and Transformer visual applications, as shown in **Table 8**. In Experiments 5, 6, and 7, when CNN and Transformer models use the Method 1 or the Method 2, model performance is generally improved compared to the original (Orgin) training method.

## 5 Conclusion and future work

In response to the challenges of difficult data collection, limited dataset size, and the diversity of plant diseases, in the context of recognition tasks using small-scale datasets, this paper proposes the use of the DDPM for data augmentation and dataset expansion. The DDPM can generate high-quality images, providing better coverage of the sample distribution compared to GANs and producing more diverse data compared to OpenCV-based augmentation techniques. In contrast to traditional data augmentation techniques such as OpenCV and GAN-based methods, DDPM diffusion model augmentation enhances the model’s generalization capabilities more effectively. Furthermore, this paper introduces a training approach using transfer learning fine-tuning. In cases of limited samples, transfer learning is applied to transfer the model’s generic features from other pre-trained networks, leading to better initial model performance, improved training convergence, and greater progressive learning. The methods proposed in this paper, Method 1 and Method 2, can be applied to various model types, including CNN and vision-based Transformers.

In future work, we will carry out the following research:

(1) To address the challenge of recognizing plant leaf disease images with high complexity, which makes training more difficult, our research team will enhance relevant model structures to cater to different needs. For example, we will explore the use of swarm intelligence techniques to optimize the Swim Transformer, thereby further improving the recognition of citrus leaf diseases and pests. We will also attempt to incorporate the ‘FreeU’ technique to enhance the DDPM diffusion model, thereby improving the quality of samples generated by the diffusion model.
(2) We will attempt to apply the research findings to plant inspection vehicles or drones. On one hand, this will enable the automated collection of field plant leaf disease datasets. On the other hand, by using improved training methods and models for image classification and object detection, our goal is to deploy them on unmanned vehicles and drones to achieve automated plant disease inspection. This, in turn, will provide support for plant disease prevention and control.
(3) We will further delve into the research of plant disease recognition, collecting datasets with a wider variety of plant species and different types of diseases, including those in peppers, grapes, apples, and more. Our aim is to transfer and apply the findings from this paper and future related work to these new datasets.

## Data availability statement

The raw data supporting the conclusions of this article will be made available by the authors, without undue reservation.

## Author contributions

YL: Writing – review & editing, Data curation, Methodology, Writing – original draft. JG: Methodology, Writing – original draft, Conceptualization, Funding acquisition, Project administration, Writing – review & editing. HQ: Writing – review & editing, Formal Analysis, Investigation, Validation, Visualization. FC: Investigation, Resources, Supervision, Visualization, Writing – review & editing. JZ: Investigation, Validation, Visualization, Writing – review & editing.
